# Segmental femoral fracture malunion: evidence and prognostic analysis of medical intervention in the third century BC

**DOI:** 10.1038/s41598-024-55300-5

**Published:** 2024-02-28

**Authors:** Haiyang Xing, Ruiqi Zou, Xiongfeng Tang, Min Yi, Zhuoting Xie, Sen You, Jianhua Liu, Quanchao Zhang, Yanguo Qin

**Affiliations:** 1 The Orthopaedic Medical Center, Second Hospital of Jilin University, Changchun, Jilin Province China; 2 School of Archaeology, Jilin University, Changchun, Jilin Province China; 3 Department of Radiology, Second Hospital of Jilin University, Changchun, Jilin Province China; 4 Jilin University, Joint International Research Laboratory of Ageing Active Strategy and Bionic Health in Northeast Asia of Ministry of Education, Changchun, China

**Keywords:** Fracture, Medical intervention, Femur, Segmental, CT, Finite element analysis, Musculoskeletal system, Archaeology

## Abstract

We examined the remains of an individual who was unearthed from the Tuchengzi site and was believed to be from the Warring States period in China. The remains exhibited segmental femoral fracture. We aimed to deduce the cause of fracture, medical interventions, healing process, and motion behavior after fracture healing using several techniques, including macroscopic observation, computed tomography (CT), and finite element analysis. Based on the morphology of the long bones, it appeared that the individual was male. The fractures resulted in an adduction angle of 5.47° and an anterior flexion angle of 21.34° in the proximal femur, while the femoral neck anteversion angle had been replaced by a retroversion angle of 10.74°. Additionally, the distal femur formed an abnormal anterior convex angle of 144.60°. CT revealed mature callus formation and visible trabecular bundles. The finite element analysis indicated that the maximum von Mises stress in the femur was 17.44 MPa during standing and 96.46 MPa during walking. We suggest that medical practitioners in the Warring States period possessed a good knowledge of thigh anatomy, enabling them to perform fracture reduction and fixation. Reasonable medical intervention facilitated fracture healing and load recovery. Satisfactory fracture healing ensured that the individual could engage in normal standing and walking activities after rehabilitation.

## Introduction

Human remains serve as the most authentic and direct evidence to aid the understanding of ancient human life^1^. They provide vital information regarding survival pressures, socioeconomic development, human ailments, and population conflicts^2^. Traumatic lesions are commonly identified when examining human skeleton remains^3^, especially traumatic lesions of the cranial bones and long bones of the limb. These lesions can be used to explore disease etiology^4,5^, social culture^6–8^, and medical advancements^3,9^ from broad cultural and social perspectives.

Although fractures are frequently encountered in human remains, there are few historical reports of the medical interventions that were used to treat fractures. Archaeologists face challenges when studying the medical treatment of fractures for several reasons. First, the splints used for fracture treatment were often constructed from wood, which is challenging to preserve. Second, the absence of soft tissue makes the details of diagnosis difficult to obtain. Finally, complete bone remodeling makes it difficult to identify fractures or ascertain the post-traumatic time interval. Although historical accounts of medical interventions for fractures in ancient times exist in the literature^9–12^, the effectiveness of these medical interventions is often inferred based solely on fracture reduction, without considering the body’s natural ability to correct displacement. Moreover, there is a lack of evidence to support the rationality of fracture treatment methods. Furthermore, these records do not reflect the advancements in the anatomical understanding of the human body and fractures. The present study provides specific evidence of the medical interventions that were used to treat fractures and detail the initial understanding of thigh anatomy by medical practitioners in China during the Warring States period.

In the present study, we investigated the remains of an individual with segmental femoral fracture. The remains were unearthed from the Tuchengzi site in Horinger, Inner Mongolia, China. Macroscopic observation, computed tomography (CT), and finite element analysis were used to reconstruct fracture formation, determine the nature of medical intervention, understand the healing process, and perform post-healing behavioral analysis. The application of finite element analysis in orthopedic biomechanics spans over four decades. It has been used for fracture mechanics analysis and to evaluate the efficacy of internal fixation^13,14^. Herein, we present the first documented case of segmental femoral fracture in the field of archaeology and provide initial evidence of the type of medical intervention that was used for fracture treatment in China during the Warring States period.

## Results

### Macroscopic observation

Based on the presence of epiphyseal closure, we determined that the individual was likely an adult. Markers of sex and age, such as the cranial and hip bones, were missing; therefore, we used the method proposed by Liu et al.^15,16^, in which two formulae that yield high success rates for femur-based sex determination were selected to determine the sex of the individual, as follows:

$$\begin{aligned} {\text{Z }} = & {\text{maximum femur length}} + 0.{\text{5312 mid}} - {\text{femur circumference}} + {6}.{\text{9691 superior femur width}} \\ & + {5}.{\text{7888femur epicondyle breadth}} - {3}.{\text{4722 lateral femoral condyle length}} \\ \end{aligned}$$

$$\begin{aligned} {\text{Z}} = & {\text{maximum femur length}} + {1}0.{96}0{\text{5 sagittal subtrochanteric femur diameter}} \\ & + {6}.{\text{9846 superior femur width}} + {7}.{\text{2925 femoral head maximum diameter}}. \\ \end{aligned}$$

**Figure 1:**
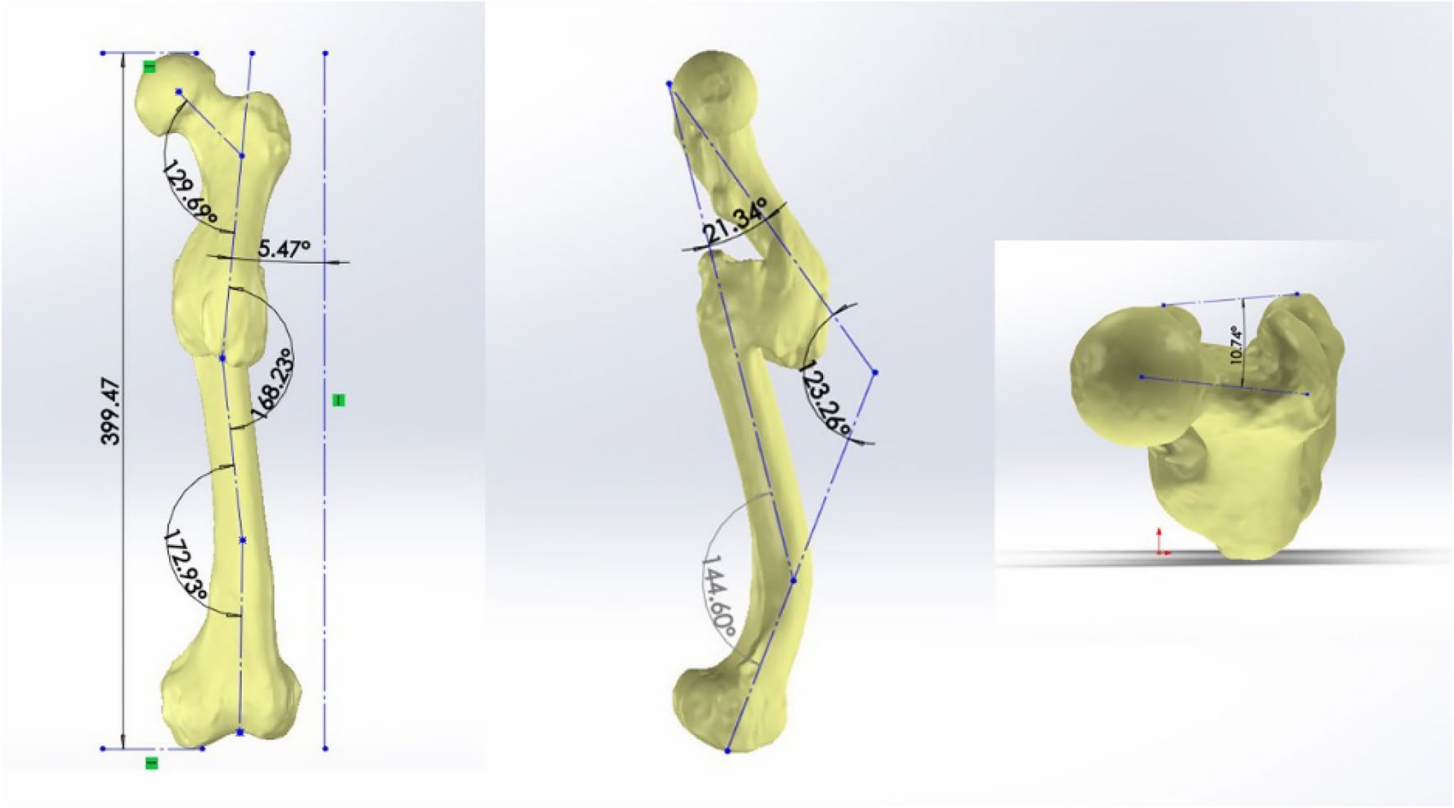
Measurement of displacement of the left femoral fracture segments in HT1M18.

Using these formulae, HT1M18 (the name used to identify the remains) was identified as male, with the left femur displaying evident signs of a fracture in the proximal one-third of the diaphysis. Displacement of the proximal fracture fragment occurred (Fig. 1). Intortion meant that the femoral neck anteversion angle disappeared and was replaced by a retroversion angle of 10.74°. The adduction angle was 5.47°, and the flexion angle was 21.34°. A conspicuous periosteal reaction was observed at the fracture site, resulting in the formation of an exaggerated bone callus. The bone callus exhibited favorable remodeling, bridging the fracture fragments and closing their medullary cavities. Simultaneously, an abnormal lordosis angle of 144.60° formed in the distal one-third of the diaphysis. No macroscopic signs of fracture or periosteal reaction were detected, and the cause of the abnormal lordosis could not be determined.

### Radiological study

**Figure 2:**
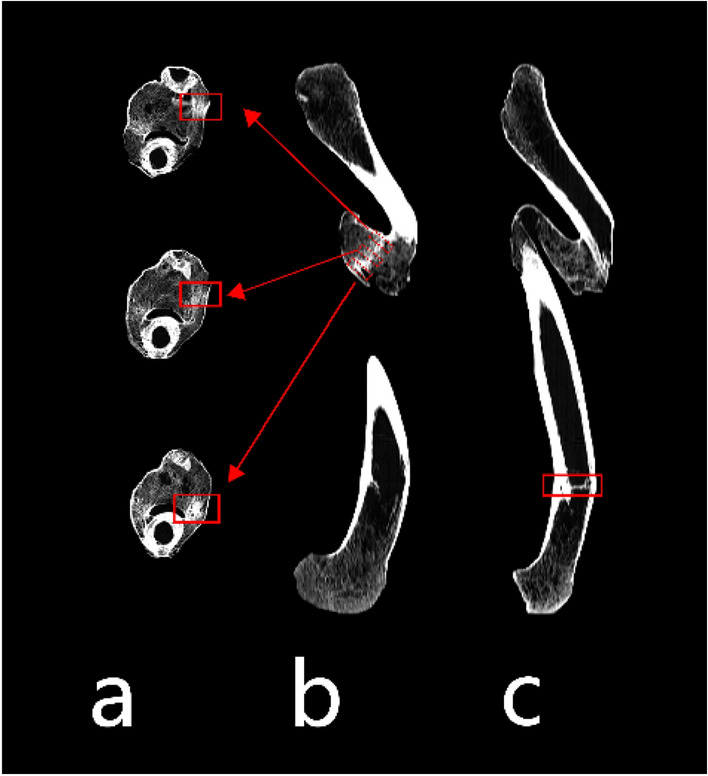
CT image of the femur. (**a** and **b**) show the proximal fracture segment callus and bundles of pressure trabeculae; (**c**) shows the distal impacted fracture segment with an intramedullary callus.

CT revealed a concurrent double femoral fracture in the proximal one-third and distal one-third of the diaphysis. The proximal fracture exhibited an oblique pattern with a notable periosteal reaction at the fracture site, giving rise to a substantial bone callus that bridged the fractured ends and sealed off the bone marrow cavity. Callus remodeling occurred, and internally, pressure-induced trabecular bundles were observed (Fig. 2a,b), indicating that the individual engaged in extensive weight-bearing activities after fracture healing. The presence of sufficient mechanical stimulation prompted bone remodeling and trabecular bundle formation^17^. Conversely, the distal fracture displayed a transverse insertion pattern, which was accompanied by bone remodeling and bone callus development within the medullary cavity (Fig. 2c).

### Finite element analysis

**Figure 3:**
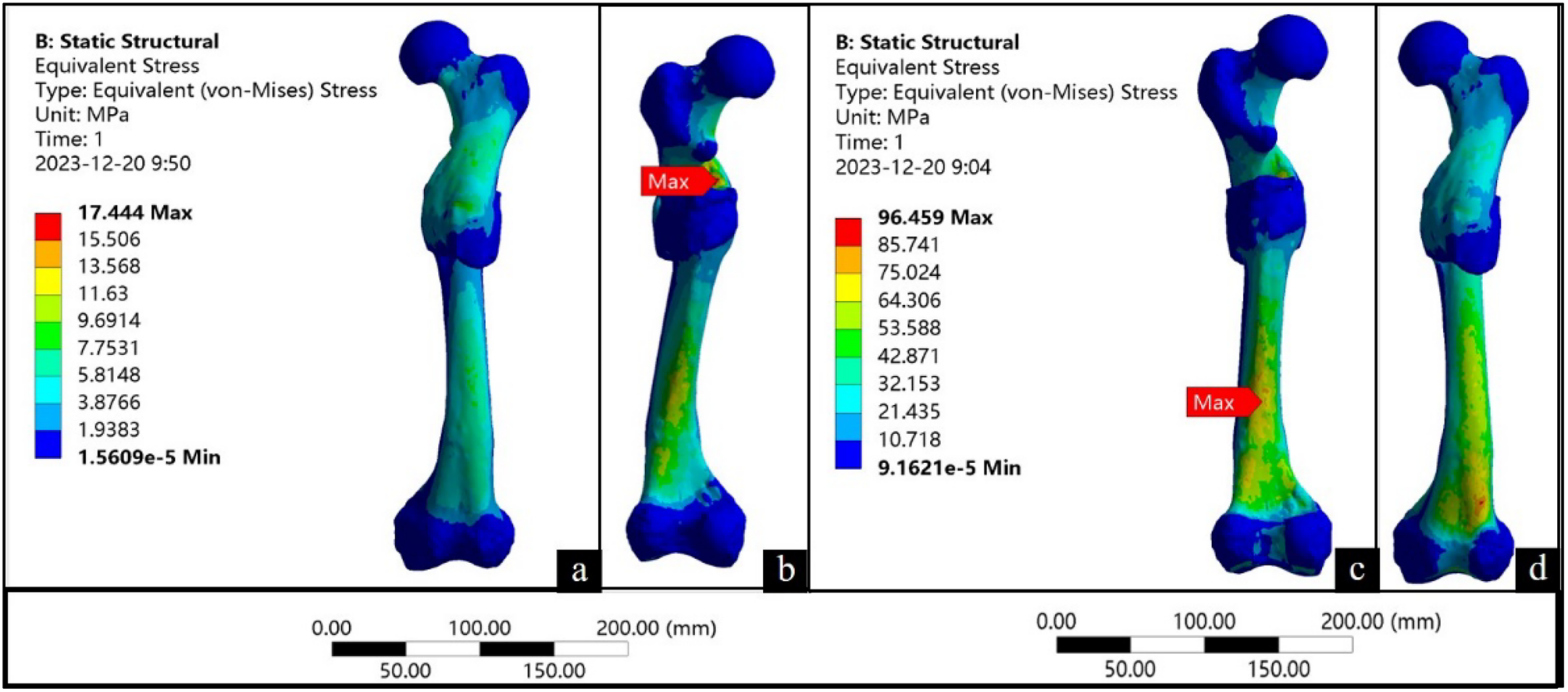
Finite element analysis results. (**a** and **b**) show the analysis when standing; (**c** and **d**) show the analysis when walking.

The finite element analysis revealed that in the standing position, the femur exhibited a localized area of concentrated stress along the medial side at the proximal end of the bone callus (Fig. 3b). The maximum von Mises stress was 17.44 MPa, while the stress distribution in other regions remained uniform (Fig. 3a,b). During walking, the stress was concentrated at the distal end of the femoral shaft, near to the distal fracture. (Fig. 3c,d). The maximum von Mises stress was 96.46 MPa.

## Discussion

Macroscopic observation and CT identified the fracture of the left femur of HT1M18 as segmental femoral fracture. The fracture was located at the proximal one-third and distal one-third of the diaphysis. We determined that the segmental fracture resulted from a single traumatic event based on the extent of bone callus formation and remodeling.

Segmental femoral fracture is regarded as a specific type of fracture with a low incidence. Such fractures are commonly observed in younger people, and they are often associated with high-impact trauma, such as traffic accidents, heavy object impacts, or falls from great heights^18–20^. However, in the present study, CT revealed that both parts of the fracture exhibited oblique and transverse patterns, while there was no indication of spiral or comminuted fracture. Consequently, we determined that the fracture was not a result of indirect force or crushing injury; rather, it was a result of direct trauma. Stress fractures and atypical fractures were ruled out based on their morphology and location^21,22^. Additionally, the absence of infection and open fractures can be inferred from bone callus formation and remodeling^11^. Numerous war-related mass graves, decapitated remains, and weapon remnants, such as arrowheads, bronze swords, and spearheads, have been discovered in several burials at the Tuchengzi site, which, in conjunction with historical documents, has been identified as a war stronghold characterized by frequent violent conflicts^23,24^. Hence, we posit that the segmental fracture in the present case resulted from either a fall from a horse or a fall from a considerable height during battle. The high prevalence of violent trauma in this region contributed to a high incidence of fracture, subsequently driving the rapid accumulation of knowledge and expertise in fracture treatment among medical practitioners, as well as advancements in medical care.

Femoral fractures, particularly segmental femoral fractures, are frequently accompanied by complications, such as hemorrhage^25,26^, fat embolism^27,28^, knee stiffness^29^, and deep vein thrombosis of the lower extremity^30^. The emergence of any of these complications poses a significant threat to this individual’s life. Therefore, the trauma experienced by HT1M18 would likely have presented a formidable challenge to the medical standards of that era. CT demonstrated pronounced bone callus formation and remodeling in the fracture. Given that remodeling after fracture takes several years in humans, it was inferred that the individual survived for at least 1 year after the fracture occurred^31^. Therefore, the fracture itself was not the direct cause of the individual’s demise. We concluded that the medical care during that time was sufficiently advanced to provide fracture fixation and high-quality treatment, thereby ensuring the individual’s survival despite severe trauma.

**Figure 4:**
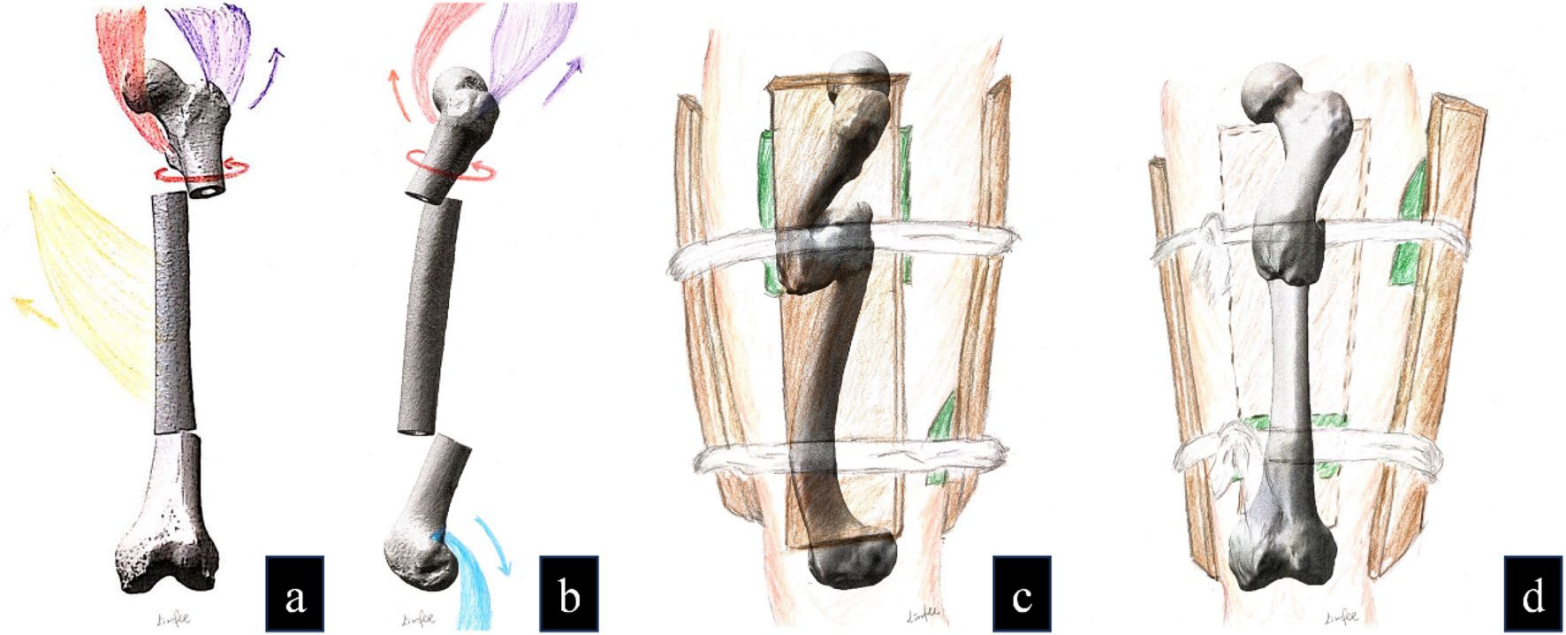
Simulation diagram of fracture displacement and fixation. (**a** and **b**) show the iliacus muscle in red, the gluteus medius muscle in purple, the hip adductor muscle groups in yellow, and the gastrocnemius muscle in blue. (**c** and **d**) depict the splints in brown and the compression pad in green.

From the perspective of fracture displacement, segmental femoral fracture typically involves three fracture segments, each of which tends to undergo displacement due to muscular traction in the absence of human intervention (Fig. 4). In the present case, the proximal fracture segment exhibited deformities in flexion, external rotation, and abduction owing to the pull exerted by the muscles (Fig. 4a,b), such as the iliopsoas, gluteus medius, and gluteus minimus muscles, along with other muscles around the hip^32,33^. The mid-segment of the fracture demonstrated adduction and outward angulation due to the tension exerted by the hip adductor muscle groups (Fig. 4a). The distal fracture segment demonstrated posterior displacement due to the traction exerted by the muscle groups surrounding the knee joint (Fig. 4b), such as the gastrocnemius muscle, as well as the gravitational force exerted on the limb^34^. However, abduction and external rotation displacement of the proximal fracture fragment and posterior displacement of the distal fracture fragment were rectified. The distal end displayed signs of overcorrection, indicating that the individual was treated with fracture reduction and splinting, with the use of compression pads beneath the splint to maintain reduction. The proximal compression pad was positioned on the anterolateral aspect of the proximal thigh to counteract muscle tension and correct the abduction and external rotation of the fracture mass (Fig. 4c,d). The distal compression pad was placed on the posterior aspect of the distal thigh to counteract muscle traction and rectify the posterior displacement of the fracture mass (Fig. 4c,d).

Segmental femoral fracture often results in significant lower-extremity shortening^35,36^, necessitating the use of lower-limb traction before fracture reduction, particularly for distal fracture segments. Thus, the individual in the present case appeared to have undergone brief lower-extremity traction before resetting, although the traction was likely not sustained, leading to femur deformity and shortening after fracture healing. Despite anatomically aligned fracture healing not being achieved, reduction of the distal fracture segment met the criteria for functional realignment. This understanding of fracture reduction using compression pads to counteract muscle tension led us to conclude that the medical practitioners of the Warring States period possessed a good knowledge of thigh anatomy, enabling them to perform appropriate fracture reduction and fixation.

From the perspective of fracture healing, the individual exhibited significant bone callus formation in the proximal one-third of the femoral fracture, indicating the application of semi-rigid fixation, which triggered endochondral bone formation. Conversely, no obvious bone callus was observed in the distal one-third of the diaphysis, implying the implementation of rigid fixation leading to direct bone formation^37^. CT revealed the presence of pressure-induced trabeculae within the bone callus. These observations indicate that the fracture underwent the three phases of fracture healing, namely inflammation, repair, and remodeling^37–39^. Previous studies have demonstrated that both early fracture instability and insufficient mechanical stimulation during the later stages can impact bone healing^37,40^. Therefore, based on the results of fracture healing, it can be inferred that medical practitioners during that era possessed rudimentary knowledge of the fracture healing process, enabling them to guide the appropriate treatment of this individual, avoiding premature weight-bearing that could impede fracture healing and complications arising from prolonged fixation.

**Figure 5:**
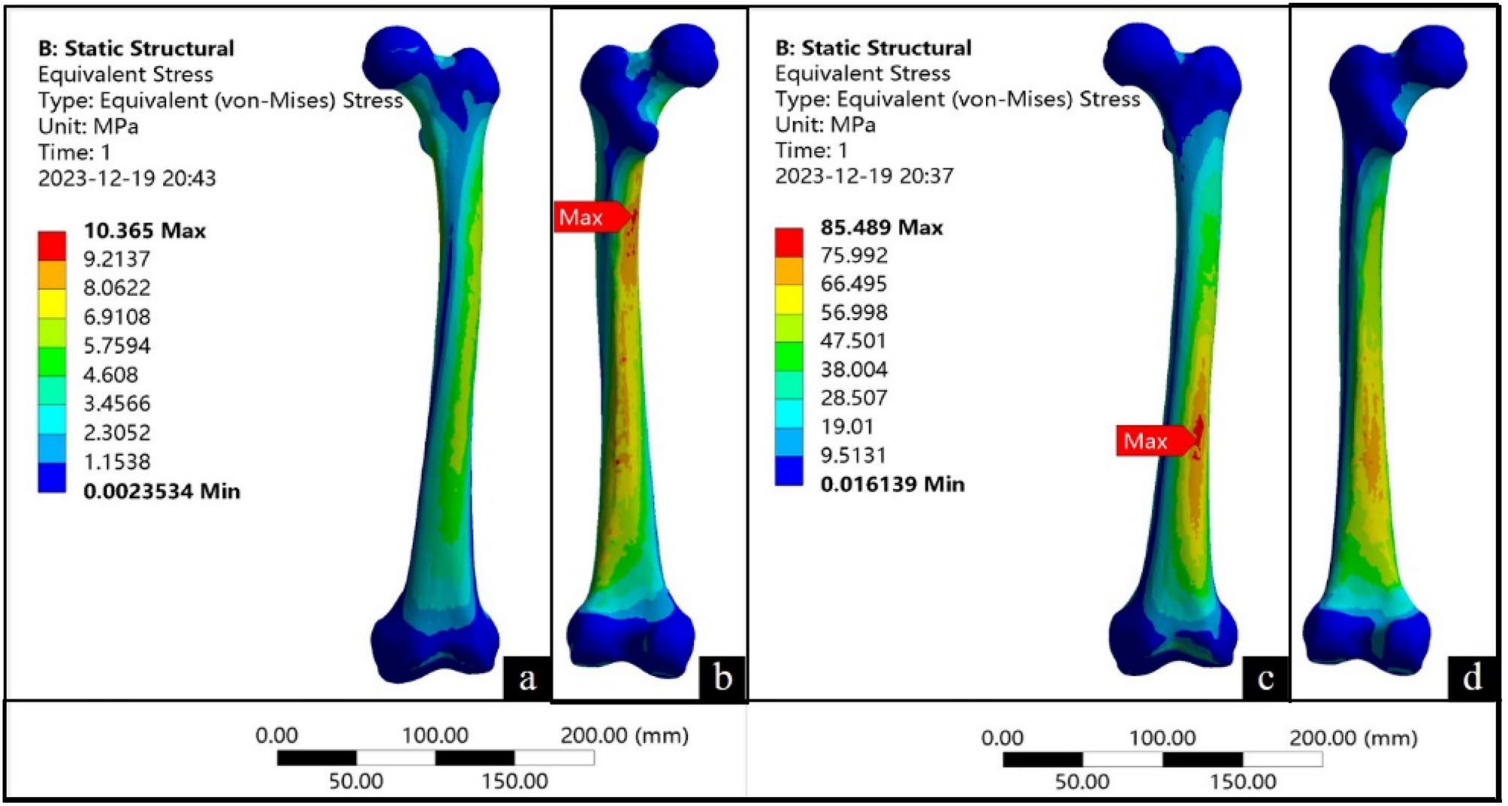
Finite element model verification.

The finite element analysis results revealed variations in the magnitude, extent, and stress distribution at the fracture site during different motion states. In the standing position, the stress was primarily concentrated medially at the proximal end of the bone callus, aligning with the direction of femur force transmission. The maximum von Mises stress was 17.44 MPa, which is significantly lower than the yield strength of the femur (120–140 MPa)^41^. This suggests that the bone callus could withstand the load experienced during standing, and the individual would have been capable of standing after fracture healing. In the walking position, the stress was concentrated near the end of the femoral shaft where the fracture occurred. This is the same location as where stress is concentrated in people without femoral fracture (Fig. 5c). The maximum von Mises stress was 96.46 MPa, which is lower than the yield strength of the femur (120–140 MPa)^41^. This means that after the fracture had healed, the individual could walk normally again. The results of the finite element analysis show that even with deformities during the healing process, satisfactory healing can still be achieved, and the normal function of the femur can be restored.

This study reports the examination results of a human specimen that was unearthed from the Tuchengzi site, Horinger, Inner Mongolia, China, which was believed to be from the Warring States period (third century BC). The individual experienced segmental femoral fracture. The occurrence of segmental femoral fracture could be directly attributed to an injury associated with an occupational activity. The medicine standards in China during the Warring States period appear to have been sufficiently advanced to successfully treat such complex fractures. We suggest that medical practitioners must have possessed a comprehensive understanding of thigh anatomy, enabling them to effectively guide fracture reduction and fixation. The application of appropriate medical intervention facilitated fracture healing and restoration of load-bearing capacity in the present case. Even though the femur was deformed, it could still function properly. This case is the first report of segmental femoral fracture within the field of archaeology, and it serves as the earliest evidence of medical intervention for fractures in China. The findings substantially contribute to our understanding of ancient Chinese medical practices, and further research endeavors are warranted to comprehensively explore the extent of medical care in ancient China.

## Methods

### Archaeological background

**Figure 6:**
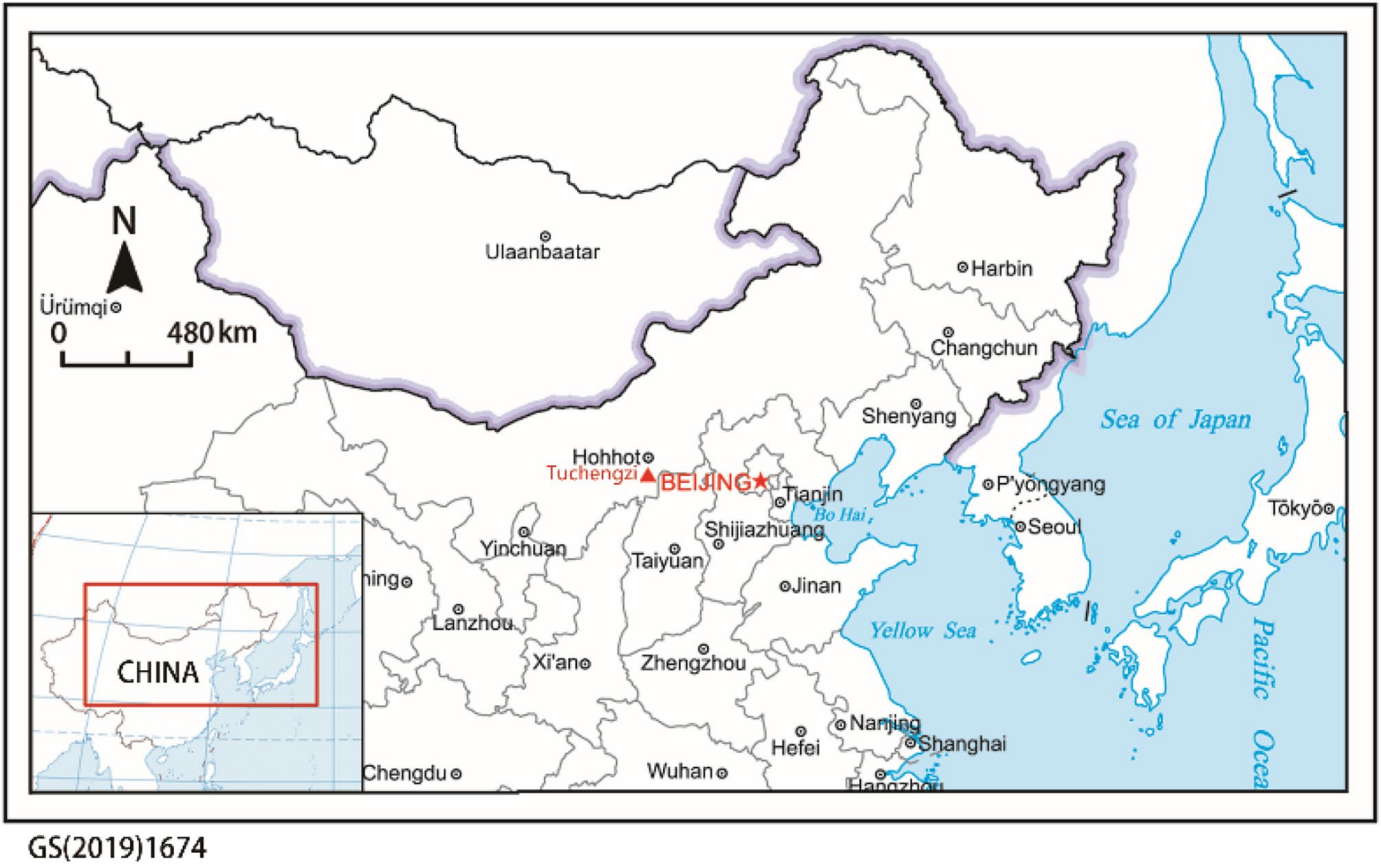
Location of the Tuchengzi site in Horinger, Inner Mongolia, China.

The Tuchengzi site, situated in Horinger, Inner Mongolia, China, at the southern base of the Yin Mountains and on the northern shore of the Yellow River, served as a significant thoroughfare from the Central Plains to the northern desert in ancient times (Fig. 6). Between 1997 and 2002, the Institute of Archaeology of Inner Mongolia Autonomous Region conducted several surveys and excavation campaigns at the Tuchengzi site and its surrounding tombs. Based on archaeological data analyses, the Tuchengzi site is broadly classified into six distinct periods (Supplementary Table S1). Archaeological investigations and historical documents^23,24^ indicate that during the Warring States period in ancient China, the state of Zhao military forces at this location and soldiers in the garrison predominantly engaged in agricultural activities.

**Figure 7:**
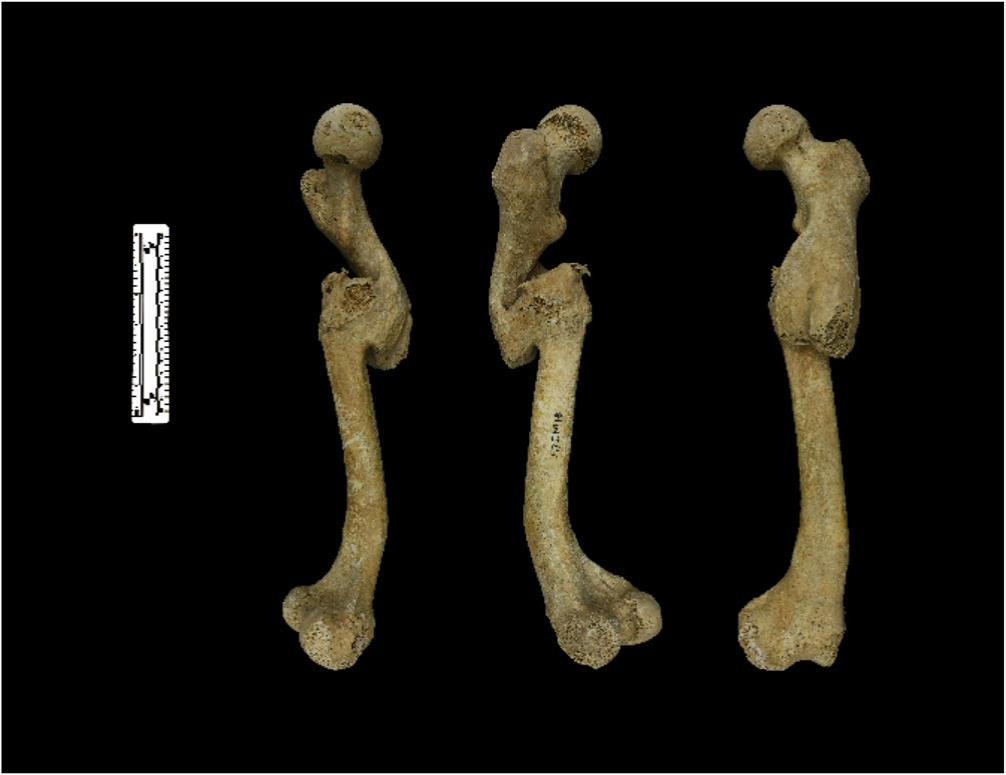
Left femur of HT1M18. The posteromedial, posterolateral, and anterior femur, respectively, are shown from left to right.

The human specimen examined in this study was identified as HT1M18 and originated from a burial dating back to the Warring States period (third century BC) at the Tuchengzi site, which was excavated between 1997 and 2002. The skeleton of HT1M18 is incompletely preserved, with only the bilateral femur, bilateral tibia, and right humerus remaining. Our examination centered on the left femur of HT1M18, wherein the proximal one-third exhibited an exaggerated bone callus connecting two angular and overlapping fracture fragments, while the distal one-third protruded abnormally (Fig. 7).

### Macroscopic observation

Macroscopic observation of all remaining bones of HT1M18 was performed following the methodology established by Roberts and Connell^42^. Morphometric measurements of the femur, tibia, and humerus were obtained by Shao’s approach^43^ (Supplementary Tables S2, S3, and S4). Markers of sex and age, such as the cranial and hip bones, were missing; therefore, the long-bone sex determination formula was utilized to determine the sex of the individual^15,16^. The body mass of HT1M18 was estimated using the body mass formula proposed by Ruff, McHenry, and Grine et al.^44^, taking the mean value calculated from the three formulae.

### Radiological analysis

CT of the left femur of HT1M18 was performed to generate a three-dimensional virtual reconstruction. Conventional medical radiological equipment (Neusoft NeuViz Glory CT, Neusoft Medical Systems, Shenyang City, Liaoning Province, China) was used with the following imaging parameters: tube current, 59 mAs; tube voltage, 140 kV; slice thickness, 0.625 mm; resolution, 512 × 512; pixel size, 0.5 mm; layers, 750.

### Finite element analysis of the femur

To extract the left femur mask from CT images in DICOM format, the first step was to import them into MIMICS Research 21.0 (Materialise Inc., Leuven, Belgium). Once uploaded, a three-dimensional model was generated using the ADVANCED SEGMENT command. Next, the model was imported into 3-matic Medical 13.0 (Materialise Inc.) and the Mark, Fix, and Local Smoothing commands were utilized to prepare the model for meshing. The Adaptive Remesh feature was used to create a triangular surface mesh with a size of 3 mm, producing a surface mesh count of 51,652 and a node count of 25,826. The Inspect Part feature was used to ensure that the surface mesh Height/Base (A) was greater than 3, indicating satisfactory mesh quality. Then, a tetrahedral 10-node volume mesh with a size of 3 mm was created using the Create Volume Mesh feature, resulting in a volume mesh count of 729,936 and a node count of 1,011,366. Once the satisfactory quality of the volume mesh had been confirmed, the three-dimensional model was imported into MIMICS Research 21.0 and assigned material properties based on its CT gray value. The gray values of the femur were evenly divided into 10 intervals. The material values of the femur were assigned according to the following formula^45–48^:

$$\begin{aligned} {\text{Density}} = & - 13.4 + 1017 \times {\text{Gray values}} \\ {\text{E}} - {\text{Modulus}} = & - 388.8 + 5925 \times {\text{Density}} \\ {\text{Poisson's ratio}} = & 0.3. \\ \end{aligned}$$

The final material parameters of the femur are shown in Table 1. The three-dimensional model of the femur was exported after material assignment.

**Table 1:** The final material parameters of the femur.

	ρ	E	v
1	153,897	9.12E + 08	0.3
2	461,718	2.74E + 09	0.3
3	769,539	4.56E + 09	0.3
4	1.08E + 06	6.38E + 09	0.3
5	1.39E + 06	8.21E + 09	0.3
6	1.69E + 06	1.00E + 10	0.3
7	2.00E + 06	1.19E + 10	0.3
8	2.31E + 06	1.37E + 10	0.3
9	2.62E + 06	1.55E + 10	0.3
10	2.92E + 06	1.73E + 10	0.3

**Figure 8:**
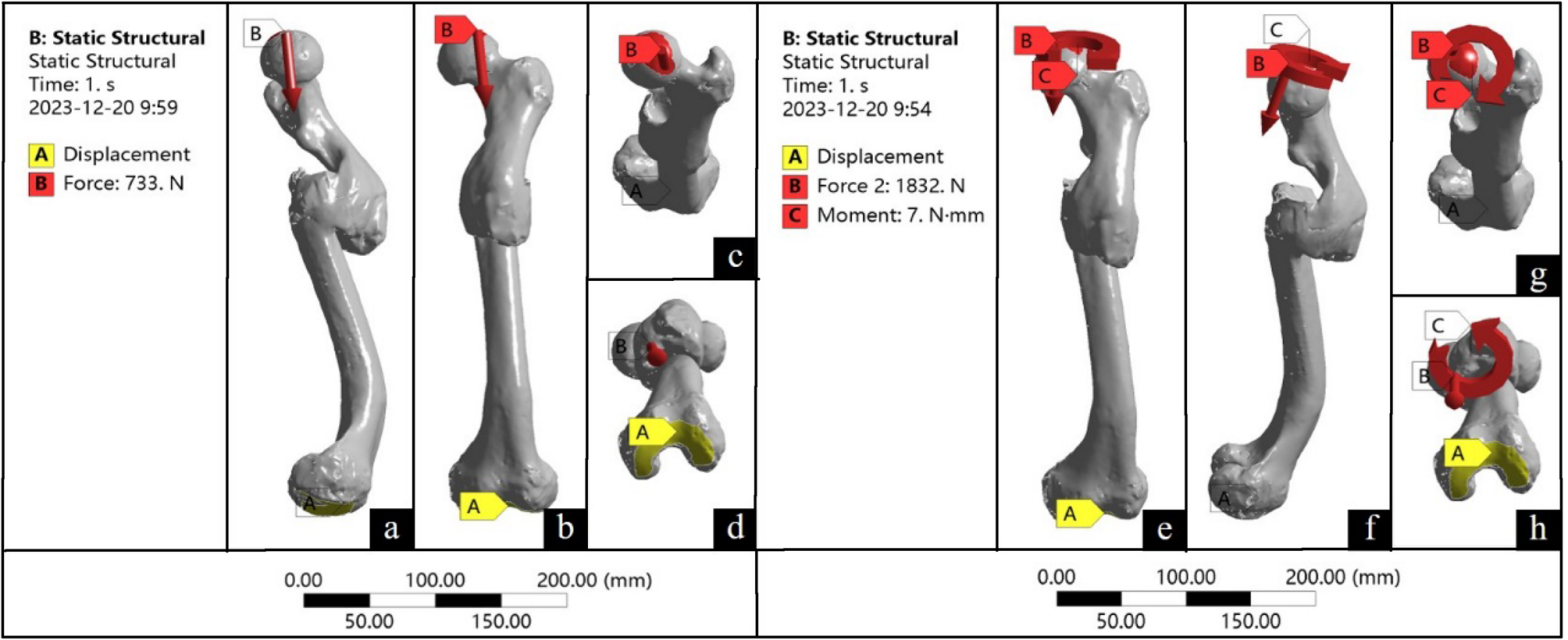
Load used in the finite element model.

The model was imported into Ansys Workbench (Ansys, US), the material properties were assigned, the coordinate system was reconstructed, load was applied to the femoral head^49^, and the distal joint surface of the femur was fixed. To simulate activity after fracture healing, standing and walking conditions were included in this study. It has been reported that during standing and walking, the stress on the hip joint is100–140% and 211–285% of body weight, respectively^50^. Additionally, the femur undergoes varying degrees of torsional force during walking^51,52^. The loads for standing and walking were set (Table 2, Fig. 8). For standing, the load was 100% of the body weight, and hip joint adduction was 15°. For walking, the load was 250% of the body weight, and hip joint adduction was 10°, flexion was 20°, external rotation was 15°, and torque was 7 N·m. The von Mises stress distribution in the femur was calculated under these two conditions.

**Table 2:** Mechanical loading of finite element analysis.

Position	Load	Adduction	Flexion	External rotation	Torque force
Standing	733 N	15°	–	–	–
Walking	1832 N	10°	20°	15°	7 N·m

### Finite element model verification

We conducted finite element analysis on a non-fractured femur by following the same steps (Fig. 5a–d). The results were similar to previous femoral biomechanical analysis results^53–55^. This demonstrates the high level of reliability and authenticity of the finite element model used in this study.

### Supplementary Information

Supplementary Tables.

## Data Availability

All data generated or analyzed during this study are available in the main text and supplementary information.
