# Supplementary material for: Segmental femoral fracture malunion: evidence and prognostic analysis of medical intervention in the third century BC
Source: Sci Rep. 2024 Feb 28;14:4930. doi: 10.1038/s41598-024-55300-5 (PMC10902296; doi:10.1038/s41598-024-55300-5)
Supplement: Supplementary file 1 — Supplementary Tables. [file 41598_2024_55300_MOESM1_ESM.docx]

**Segmental Femoral Fracture Malunion: Evidence and Prognostic Analysis of Medical Intervention in the 3rd Century BC**

Xing H Y^1^, Zou R Q^2^, Tang X F^1^, Yi M^1^, Xie Z T^1^, You S^2^, Liu J H^3^, Zhang Q C^2，*^, Qin Y G^1，*^

1 The Orthopaedic Medical Center, Second Hospital of Jilin University, Changchun, Jilin Province, China

2 School of Archaeology, Jilin University, Changchun, Jilin Province, China

3 Department of Radiology, Second Hospital of Jilin University, Changchun, Jilin, Province, China

*Corresponding authors

E-mail address of the corresponding author: qinyg@jlu.edu.cn (Qin Y G), py2000sdqy@sina.com (Zhang Q C)

| Chinese dynasties | Number of tombs |
| --- | --- |
| Spring and Autumn period | 10 |
| Warring States period | 1,030 |
| Qin and Han Dynasties | 359 |
| Wei and Jin Dynasties | 51 |
| Tang Dynasty | 198 |
| Liao, Jin, and Yuan Dynasties | 5 |

**Supplementary Table S1.** Dynasty and number of tombs in and around the Tuchengzi site

| Item | Male | | Female | | This sample | |
| --- | --- | --- | --- | --- | --- | --- |
|  | L | R | L | R | L | R |
| Femur maximum length | 434.28 | 435.35 | 400 | 400 | 399.47 | 448.42 |
| Femur physiological length | 429.9 | 430.81 | 394.8 | 394.82 | 398.1 | 444.55 |
| Femur transverse subtrochanteric diameter | 32.75 | 32.93 | 29.32 | 29.06 | — | 33.38 |
| Femur sagittal subtrochanteric diameter | 25.41 | 25.6 | 21.77 | 22.19 | — | 24.76 |
| Transverse diameter of femoral shaft | 27.67 | 27.36 | 25.26 | 24.45 | — | 28.71 |
| Sagittal diameter of femoral shaft | 29.4 | 29.14 | 25.1 | 25.11 | — | 31.21 |
| Femur Midshaft Circumference | 91.16 | 91.32 | 80.9 | 79.94 | — | 92.47 |
| Minimum diameter of femur diaphyseal | 30.29 | 30.29 | 26.35 | 26.29 | 32.65 | 31.67 |
| Transverse diameter of femur diaphyseal | 39.42 | 39.54 | 35.64 | 36.1 | 38.72 | 38.12 |
| Femoral head-neck axis length | 74.77 | 75.33 | 68.13 | 66.88 | 72.58 | 75.85 |
| Vertical diameter of femoral neck | 33.69 | 34.74 | 28.71 | 28.84 | 33.21 | 33.46 |
| Sagittal diameter of femoral neck | 25.69 | 26.58 | 22.04 | 23.18 | 25.06 | 27.95 |
| Vertical diameter of femoral head | 45.1 | 44.95 | 39.69 | 39.25 | 47.3 | 45.13* |
| Sagittal diameter of femoral head | 46.08 | 46.35 | 41.09 | 40.86 | 48.27 | 48.08* |
| Femur maximum head diameter | 147.25 | 149.19 | 129.58 | 131.33 | 152 | 149.99* |
| Femur epicondylar breadth | 79.06 | 80.1 | 68.23 | 69.2 | — | 83.34 |
| Length of lateral femoral condyle | 61.73 | 62.44 | 54.46 | 54.97 | 62.89* | 62.65 |
| Length of medial femoral condyle | 62.55 | 62.22 | 54.61 | 55.29 | 64.84* | 64.14 |
| Femoral neck-shaft angle | 133.03 | 134.14 | 134.42 | 137.63 | 129.69 | 132.28 |

**Supplementary Table S2.** Comparison table between the average measurement value of the femur male and female at the Tuchengzi site in the Warring States period and the measurement value of this sample (unit: mm) (" - "means that it cannot be measured, and" * "means the residual length).

| Item | Male | | Female | | This sample | |
| --- | --- | --- | --- | --- | --- | --- |
|  | L | R | L | R | L | R |
| Tibia maximum length | 358.25 | 357.41 | 325.83 | 327.08 | 370 | 369 |
| Tibia physiological length | 336.75 | 336.68 | 306.67 | 308.33 | 364 | 360 |
| Tibia midshaft maximum diameter | 29.67 | 30.19 | 25.68 | 26.21 | 30.29 | 30.02 |
| Tibia midshaft transverse diameter | 21.71 | 21.65 | 18.42 | 18.75 | 22.12 | 21.08 |
| Tibia sagittal diameter at the nutrient foramen | 34.79 | 34.72 | 29.77 | 29.92 | 35.12 | 34.65 |
| Tibia transverse diameter at the nutrient foramen | 23.19 | 23.62 | 20.33 | 20.46 | 23.05 | 22.45 |
| Proximal epiphyseal breadth of the tibia | 74.17 | 74.75 | 63.5 | 63.75 | — | — |
| Distal epiphyseal breadth of the tibia | 51.65 | 52.01 | 46.31 | 46.55 | — | 53.3 |
| Distal epiphyseal sagittal diameter of the tibia | 37.05 | 37.3 | 33.01 | 33.65 | — | — |
| Minimum circumference of the tibia shaft | 80.19 | 80.86 | 71 | 69.67 | 75 | 74 |

**Supplementary Table S3.** Comparison table between the average measurement value of the tibia of male and female at the Tuchengzi site in the Warring States period and the measurement value of this sample (unit: mm) (" - "means that it cannot be measured).

| Item | Male | | Female | | This sample | |
| --- | --- | --- | --- | --- | --- | --- |
|  | L | R | L | R | L | R |
| Humerus maximum length | 311.91 | 315.78 | 283.38 | 286.55 | 315 | — |
| Humerus physiological length | 307.24 | 311.16 | 279 | 281.91 | — | — |
| Maximum diameter of humeral midshaft | 22.16 | 22.49 | 19.65 | 19.17 | 23.47 | — |
| Minimum diameter of humeral midshaft | 18.09 | 18.03 | 15.39 | 15.04 | 17.24 | — |
| Minimum circumference of humeral shaft | 68.27 | 69.24 | 60.38 | 59.42 | 62 | — |
| Circumference of humeral head | 137.39 | 140.86 | 123.33 | 123 | — | — |
| Humeral breadth of proximal epiphysis | 48.54 | 49.44 | 43.67 | 42.69 | — | — |
| Humerus epicondylar Breadth | 61.17 | 61.58 | 52.98 | 51.91 | — | — |
| Humeral trochlea and capitellum width | 43.61 | 44.76 | 38.71 | 38.4 | — | — |
| Sagittal diameter of humeral trochlea | 26.61 | 26.48 | 22.57 | 22.4 | — | — |
| Transverse diameter of humeral head | 41.57 | 42.32 | 36.69 | 36.7 | 47.98 | — |
| Vertical diameter of humeral head | 32.04 | 31.88 | 29.29 | 29.42 | — | — |

**Supplementary Table S4.** Comparison table between the average measurement value of the humerus of male and female at the Tuchengzi site in the Warring States period and the measurement value of this sample (unit: mm) (" - "means that it cannot be measured).
